# Isolated common femoral artery aneurysm: a case report

**DOI:** 10.1186/1757-1626-2-7522

**Published:** 2009-05-08

**Authors:** Saurabh Sharma, Sanjay Nalachandran

**Affiliations:** Department of General Surgery, Tan Tock Seng HospitalJalan Tan Tock, Singapore-308433

## Abstract

**Introduction:**

Isolated aneurysm of common femoral artery is a rare occurrence. They may mimic other common conditions like groin lymph nodes or groin hernia.

**Case presentation:**

Here we present a case of 61-years-old Chinese gentleman who presented with a right groin lump, which was suspected to be groin hernia but turned out common femoral artery aneurysm. The aneurysm was surgically excised and a prosthetic vascular repair was done.

**Conclusion:**

Isolated common femoral artery aneurysms are rare and can mimic groin hernias, so one must be careful. Color Doppler is the investigation of choice. Best treatment modality is surgical excision with repair with or without graft for aneurysms > 2.5 cm.

## Introduction

Aneurysms can occur in almost any artery in the body, but the most common locations are in the abdominal aorta, thoracic aorta, cerebral vessels, and iliac, popliteal, and femoral arteries in descending order of frequency.

Femoral arterial aneurysms (FAAs) are uncommon aneurysms that are usually diagnosed as a pulsatile mass in the groin. They occur in elderly men, often with other manifestations of atherosclerosis. One third are bilateral and nearly two thirds are associated with aneurysms elsewhere (e.g., popliteal and aortic).

Isolated true femoral artery aneurysms are relatively rare and are found in elderly men with a strong history of smoking [1].

Femoral aneurysms can reach a large size and via thrombosis can create symptoms such as limb-threatening ischemia, embolization, or tissue loss; however, rupture is rarely encountered. FAAs are often asymptomatic, but local pain, distal embolization, rupture, and venous compression may all be presenting features.

Review of literature shows that femoral aneurysm after groin hernia surgery has not been reported.

Here we present a case of isolated common femoral artery aneurysm.

## Case Presentation

A 61-years-old Chinese male with past medical history of diabetes mellitus, cerebrovascular event, chronic smoking and hyperlipidemia, presented with right groin lump for one year. Previously (five years prior this presentation) the patient had similar right groin lump which was diagnosed as right inguinal hernia and he was operated on. Post operative period was uneventful and he recovered well.

At present, there was no associated pain or any other symptoms with this lump. There was no history of IV drug abuse or any groin trauma.

The patient presented initially with a left inguinal lump. Physical examination showed left inguinal hernia and right side was normal. A routine ultrasound of bilateral groin regions was ordered as part of workup for groin hernia.

Ultrasound (US) showed a 3.7 × 3.2 × 2.3 cm saccular aneurysm arising from the posterior aspect of the right common femoral artery (CFA) with thickened wall measuring 0.9 cm. It appeared to abut the common femoral vein, which was just medial. On the left side, there was inguinal hernia, accentuated with the Valsalva maneuver.

Duplex study confirmed the CFA aneurysm. The proximal segments of both the superficial femoral and profunda femoris arteries were patent and with normal lumen dimension. Triphasic flow was demonstrated.

In view of the size of the aneurysm, operation was scheduled. Per-operative findings showed a 3 × 4 × 3 cm isolated right CFA aneurysm just below right inguinal ligament. Size 10 polytetrafluoroethylene interposition graft repair was performed.

Post-operative period was uneventful. Distal pulses were intact. Patient went home on post-operative day 3.

Histology of the resected aneurysm showed it to be a true aneurysm with thrombus in lumen. There was intimal ulceration with cholesterol clefts and foamy histiocytes and plasma cell suggestive of athereosclerotic changes.

## Discussion

Primary femoral aneurysms are uncommon and are frequently associated with other aneurysms, particularly those of the aorta and popliteal arteries. True isolated atherosclerotic aneurysm of the superficial femoral artery is a rare pathology [2].

Rigdon et al discussed 17 “arteriosclerotic” superficial femoral artery aneurysms in 14 patients, which revealed complication at presentation in 65% - rupture in 35%, thrombosis in 18%, and distal emboli in 12%. However, limb salvage was 94% and there were no perioperative deaths [3]. Males (75%) were more common than females, and the average age was 77 years (range 61 to 93) [3].

True femoral artery aneurysms are attributed to weakening of the arterial wall due to atherosclerosis. True femoral artery aneurysms are relatively rare and are found in elderly men who have strong smoking history. Aortic aneurysms are approximately 10 times more common. According to Levi et al distal embolization occurs in 0-26% of cases, acute thrombosis occurs in around 15% of cases. Rupture is uncommon and varies between 10% and 14% [1].

Another review by Fluckiger et al shows the risk of complications such as rupture, thrombosis and embolization to be above 50%. In their report acute dilatation and rupture occurred in 34.5% [4]. This number emphasizes the need of a complete angiological investigation in patients with aneurysms [4].

Color duplex is the investigation of choice. Its advantages are low cost, utility in various treatment choices, no need for puncture/contrast and easy availability and use. In addition to diagnosis, it gives detailed information about the aneurysms i.e. dimensions, morphology, neck anatomy, flow and relation with adjacent vessels. Vasquez et al has emphasized on the great latency of the disease and the high incidence of complication at presentation, as well as, advantage of color duplex as diagnostic and therapeutic modality over angiography [5,6].

Femoral aneurysm after groin procedures like cardiac catheterization (0.1-5%) [7], vascular reconstruction 1-10% [8] is quite common, but not reported after hernia repair.

Complications of open inguinal hernia repair, such as wound infection, hematoma, seroma, and neuralgia, are known to occur. Vascular injuries during inguinal hernia repair are rare and documented as case reports only.

Vascular malformations are known to occur after trauma or sharp penetrating injuries. Agrawal et al has reported a case of iatrogenic venovenous malformation (VVM) in the subcutaneous space of the left inguinal region following an open inguinal hernia repair. This VVM presented with a spontaneous rupture leading to widespread ecchymosis of the thigh and was successfully managed endoscopically [9].

Teodorescu et al has reported a case with a ruptured infected iliac artery pseudoaneurysm two weeks after ipsilateral inguinal hernia repair. Pseudoaneurysms that occur because of infection develop rapidly and mandate ligation of the affected artery or extra-anatomical bypass. Noninfected pseudoaneurysms are usually discovered incidentally and may be managed with either endovascular or standard surgical techniques [10].

Injuries to the femoral vein may be caused by suture of the anterior wall of the vein during inclusion of the shelving edge of Poupart’s ligament in the repair or by compression of the femoral vein by a suture that is placed too laterally on the ligament of Cooper. These situations have complicated the Cooper ligament repair too frequently (0.35-1.6%) and have contributed to a lessened popularity of this technique.

Injury to the femoral artery can also occur with open inguinal herniorrhaphy. This may occur during reconstruction of the posterior inguinal wall near the deep inguinal ring, a site where the iliofemoral artery is situated 1 cm to 1.5 cm deep to the transversalis fascia.

All control of bleeding must be done under direct vision. All deep suture ligatures and blind clamping must be proscribed. Careful postoperative observation must be instituted for early detection of vascular impairment and its subsequent complications of thrombosis, embolization, and gangrene. Subsequent and delayed complications manifest as stenosis, false aneurysm or arteriovenous fistula [10].

Femoral artery aneurysms (FAA) can reach a large size and via thrombosis can create symptoms such as limb-threatening ischemia, embolization or tissue loss; however, rupture is rarely encountered. FAAs are often asymptomatic, but local pain, distal embolization, rupture, and venous compression may all be presenting features.

Surgery is considered the gold-standard treatment, although is not without risk in patients with severe cardiovascular disease. All aneurysms greater than 2.5 cm should be treated with resection and interposition grafting. Prosthetic graft (8- to 10-mm Dacron or ePTFE) can be used in most cases, although reversed saphenous vein graft should be used for mycotic/infected aneurysms.

## Conclusion

In conclusion, we present a case report of an isolated common femoral artery aneurysm as unusual groin lump. Duplex scan is the investigation of choice. Surgery is recommended in aneurysm greater then 2.5 cm in view of high rate of complications. The recommended technique is interposition or inlay graft and the outcome is excellent.

## List of abbreviations

CFA: Common femoral artery
FAA: Femoral arterial aneurysms
US: Ultrasound
VVM: Venovenous malformation
